# Minimally invasive treatment of tibial pilon fractures through arthroscopy and external fixator-assisted reduction

**DOI:** 10.1186/s40064-016-3601-7

**Published:** 2016-11-05

**Authors:** Huasong Luo, Liaobin Chen, Kebin Liu, Songming Peng, Jien Zhang, Yang Yi

**Affiliations:** 1 Department of Orthopedics, Zhongnan Hospital of Wuhan University, Wuhan, 430071 China; 2 Department of Orthopedics, Jingzhou First People’s Hospital, The First Affiliated Hospital of Yangtze University, Jingzhou, 434000 China

**Keywords:** Arthroscopy, External fixator, Minimally invasive surgery, Tibial pilon fracture

## Abstract

**Objective:**

The aim of this study was to evaluate the clinical outcome of tibial pilon fractures treated with arthroscopy and assisted reduction with an external fixator.

**Methods:**

Thirteen patients with tibial pilon fractures underwent assisted reduction for limited lower internal fixation with an external fixator under arthroscopic guidance. The weight-bearing time was decided on the basis of repeat radiography of the tibia 3 months after surgery. Postoperative ankle function was evaluated according to the Mazur scoring system.

**Results:**

Healing of fractures was achieved in all cases, with no complications such as severe infection, skin necrosis, or an exposed plate. There were 9 excellent, 2 good, and 2 poor outcomes, scored according to the Mazur system. The acceptance rate was 85%.

**Conclusion:**

Arthroscopy and external fixator-assisted reduction for the minimally invasive treatment of tibial pilon fractures not only produced less trauma but also protected the soft tissues and blood supply surrounding the fractures. External fixation could indirectly provide reduction and effective operative space for arthroscopic implantation, especially for AO type B fractures and partial AO type C1 fractures.

## Background

Tibial pilon fractures affect weight-loading on the articular surface and metaphysis; these severe injuries comprise about 5–10% of all lower limb fractures, and have high complication rates (range 11.4–54%) (McFerran et al. 1992). The characteristics of pilon fractures include a variable degree of metaphyseal compression, comminution, unstable fracture height, and primary injury of the articular cartilage; pilon fractures are caused by high-energy injuries and sometimes occur in combination with a severe soft tissue injury (Watson et al. 2000; Huebner et al. 2014; Schweigkofler et al. 2015; Klaue and Cronier 2015, Krettek and Bachmann 2015a, b). Pilon fractures are usually the result of combined compressive and shear forces, and may result in instability of the metaphysis. The complexity of the injury, lack of muscle cover, and poor vascularity make these fractures difficult to treat. The risk of complications was reported to be as high as 37–54% with open reduction techniques (Teeny and Wiss 1993). Surgical treatment of pilon fractures includes several options: intramedullary nailing, external fixation, and minimally invasive plate osteosynthesis (MIPO). The management of tibial pilon fractures with MIPO enables the preservation of soft tissue and the remaining blood supply.

The surgical fixation method is determined according to the extent of the fracture and displaced articular surface, as well as the condition of the soft tissue. For the treatment of pilon fractures, Watson emphasized that minimally invasive separation of soft tissue not only protects the blood supply of the fractured bone but also provides indirect reduction. Watson suggested choosing the surgical approach on the basis of the condition of the injured soft tissue, and recommended the use of limited exposure and stabilization with small wire circular external fixators.

Thirteen patients with tibial pilon fractures underwent definitive treatment with limited open reduction and internal fixation with external fixators under arthroscopic guidance at our hospital from November 2010 to December 2013. All 13 patients reported satisfaction with the clinical outcome.

## Methods

### General data

**Fig. 1:**
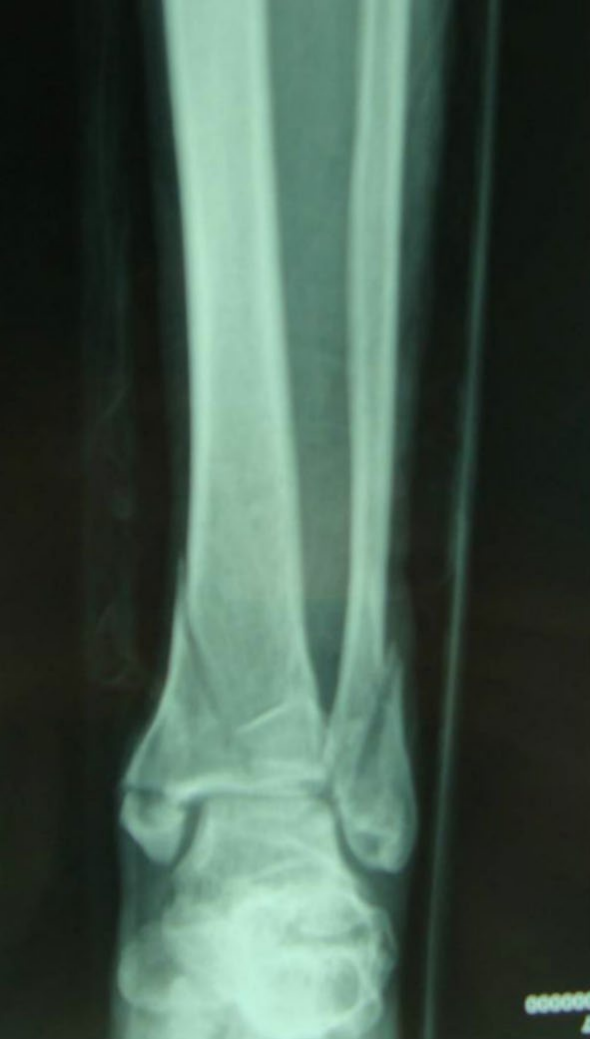
A 46 male patient had right closed pilon fracture caused by trauma (AO C1) preoperative X-ray result of right ankle joint

Thirteen patients were enrolled in this study, including 9 males and 4 females. Six patients experienced a fall from a high place, and 7 were injured in a traffic accident. Patient age ranged from 19 to 54 years. All had closed injuries (AO type B2 or C1); Fig. 1 lists the classic cases. The mean time from injury to surgery was about 10 days.

### Preoperative preparation

Physical examination was conducted in detail before surgery to determine overall status. Fractures of the femoral neck and spinal cord and closed injuries of the chest, abdomen, and brain were excluded. The ankle joint and surrounding soft tissue were evaluated for vascular status (especially the dorsalis pedis pulse) and neurologic injury. Radiological examination of the ankle and tibiofibular joints was performed routinely. The unaffected contralateral areas were also photographed as controls. Computed tomography with three-dimensional evaluation of the ankle joint was performed to determine the position of the fracture, the position and number of cortical bones, the degree of comminution, and the extent of fracture compression and displacement. Longitudinal traction was performed to restore the ankle axes as soon as possible, followed by ankle joint fixation with a plaster slab; tuberis calcanei traction was performed on patients with severe soft tissue injury. Intravenous mannitol and dexamethasone were used before surgery to alleviate local soft tissue edema and inflammation. Blisters were aspirated with a sterile syringe. Surgery was performed 7–14 days after the injury when soft tissue had healed or swelling had dissipated.

### Operative treatment

Surgical treatment was successfully used for open fractures, compartment syndrome, step-off height from the joint surface >2 mm, and tibial pilon fractures in which the angle of any plane surface was >10°. The aim of surgery was to restore the function of the bone and soft tissues, perform anatomical reduction of the joint surface, and provide stability of the fracture to allow return to functional exercise as early as possible. In selecting treatment, the extent of soft tissue injury should be considered. For a classic AO type C1 pilon fracture, we selected a minimally invasive treatment (Fig. 1). The fibula was plated and the tibial side was stabilized with a locking plate and screws. Surgical treatment was performed in multiple steps, including restoration of lower limb length, restoration of the metaphyseal outline, bone grafting, and metaphyseal reduction and fixation.

**Fig. 2:**
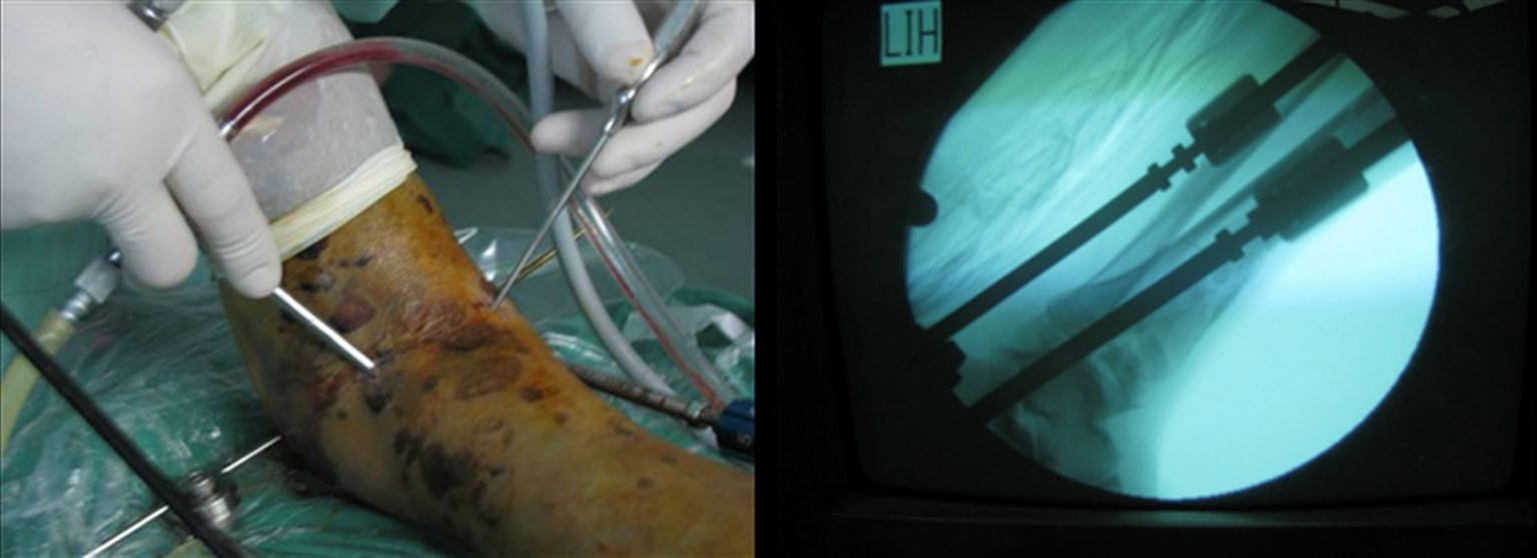
External fixator assisted reduction with implantation of the arthroscopy and monitored by medical imaging during the surgery

**Fig. 3:**
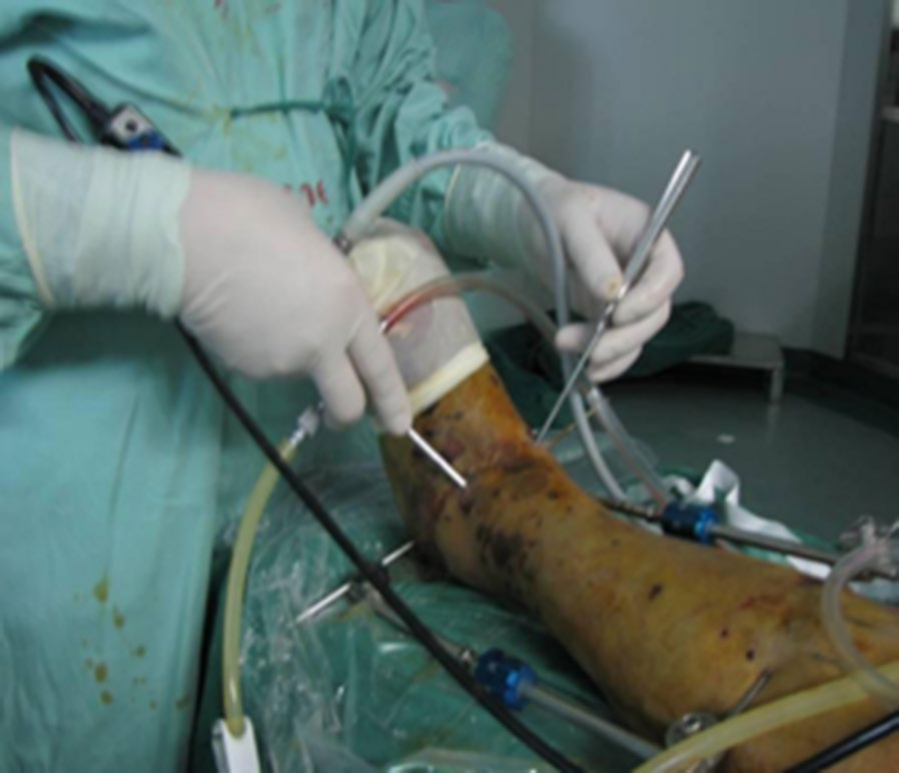
The image of the arthroscopic implantation

**Fig. 4:**
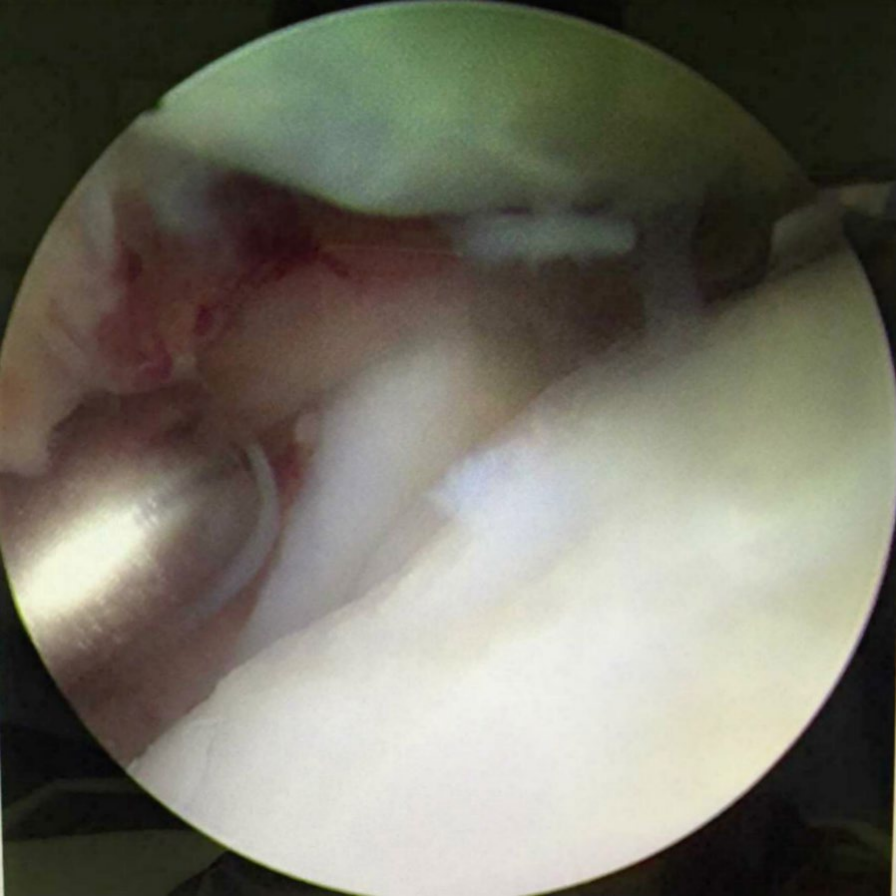
Arthroscopic views show the situation of tibial articular surface with out-of-flatness

The first step restored the length of the fibula. Posterolateral incision over the fibula was performed to expose the broken end under direct vision, followed by anatomical plate fixation after reduction. The second step involved placement of external fixators for the reduction of the distal tibia. Two screws traversed the tibia and one screw traversed the tuberis calcanei; the two sides of the distal tibia were slowly unfolded. Indirect reduction with external fixators was then performed, with an increase in soft tissue tension and recovery of limb length and alignment. The unfolding tibial reduction expanded the space in the ankle joint available for implantation and arthroscopic surgery, and allowed direct observation of the status of the reduction. The third step involved percutaneous reduction by leverage under arthroscopic guidance; this achieved planarization of the joint surface as confirmed with imaging during surgery (Fig. 2). First, two small incisions were made at the front of the ankle; then, the arthroscopic camera and instruments (Stryker Corporation, USA) were smoothly inserted into the ankle through these portals for observation and reduction of the fracture (Figs. 3, 4). The anterolateral approach was located at the outside articular line of the extensor digitorum pedis longus tendon. The anteromedial approach was located at the inside articular line of the anterior tibial tendon. Blood clots and bone scraps were cleared under arthroscopy, and the condition of fracture displacement was observed. Common fractures were directly treated with percutaneous reduction by leverage. Articular surfaces were restored to smoothness through picking with a hook. For comminuted metaphyseal fractures, a 3–4-cm longitudinal incision was made over the tibia near the joint surface; blunt dissection was then performed to reach the fracture point, and the broken bone in front of the metaphyseal fracture was exposed. A fascia stripper was used to perform percutaneous reduction by leverage of the exposed fracture pieces under arthroscopy. When the step-off height of the joint surface was <2 mm, a 1.5-mm Kirschner wire was used to temporarily fix the fracture. The fourth step involved bone grafting of the tibial metaphysis. Autogenic ilium packing traction and reduction were performed through the anterior tibial incision. The remaining bone defect in the metaphysis was subjected to adequate bone grafting. The fifth step involved tibial internal or anterior plate fixation. The use of anterior or internal anatomical plates depended on the type of fracture. A minimally invasive plate was placed between the fascia and periosteum, and 3–4 screws were used to fix the two ends of the fractures. Lag screws were needed for patients with an accompanying fracture of the posterior malleolus.

For postoperative management, antibiotics were used for 3 days to prevent infection, and the affected limb was elevated. If tolerated, the patients performed bed exercises. To determine weight-bearing time, radiography of the ankle joint was repeated at 3 months after surgery.

## Results

**Fig. 5:**
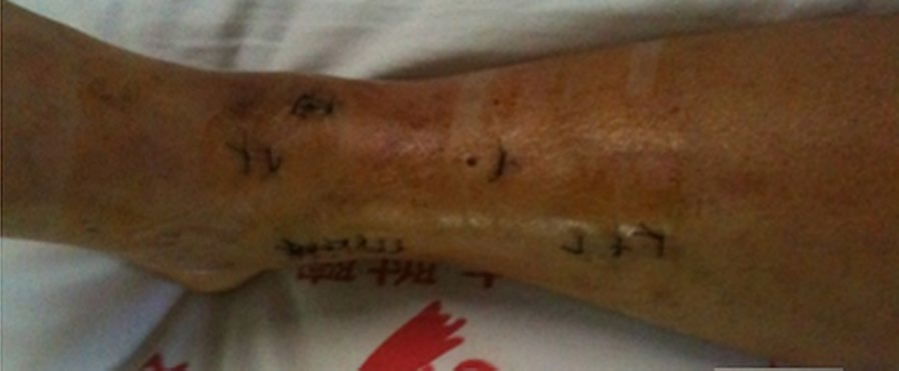
Small incision and no skin necrosis after the surgery

**Fig. 6:**
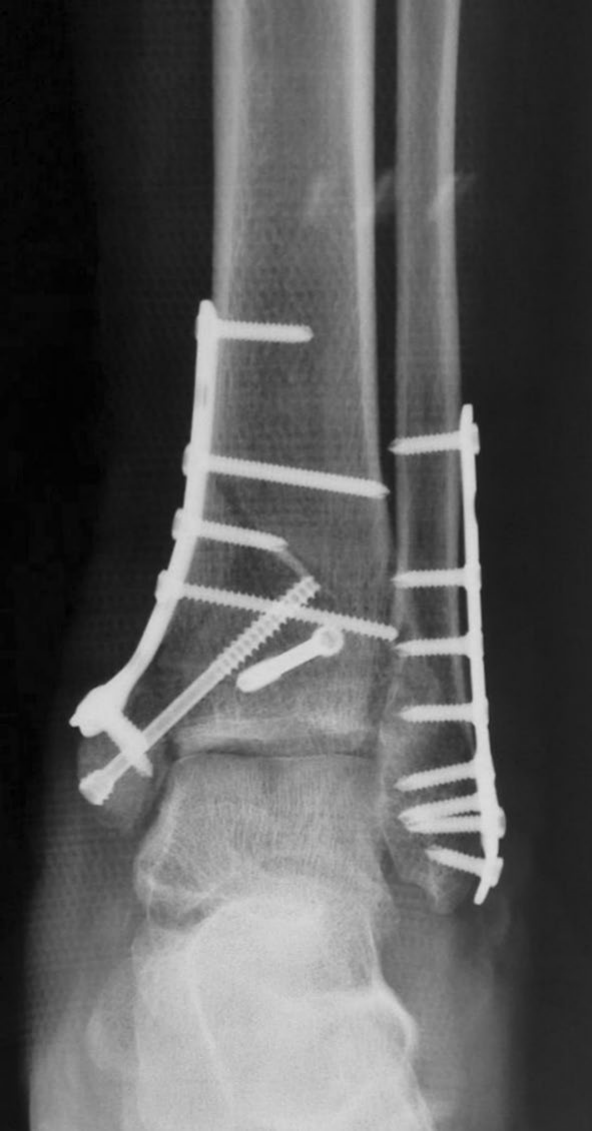
X-ray showed that inferior segments of right tibia and fibula had good reduction and the joint surface also recovered well after the surgery

Sutures were removed at 2 weeks after surgery in all patients. There were no complications such as severe infection, skin necrosis, or an exposed plate (Table 1; Fig. 5). The postoperative radiographs showed good fracture reduction and position of the internal fixators (Fig. 6). All patients were followed. Radiographic examination was performed at 3 and 6 months after surgery. The longest follow-up time was 1 year. After 6 months, the radiographic results showed good fracture union without malunion, and good callus growth in the grafted region. The fracture healing time ranged from 8 to 16 weeks (mean, 12 weeks), and no disunion occurred. The efficacy of surgery was evaluated by using the Mazur grading system, with 9 cases scored as excellent, 2 as good, and 2 as poor (Mazur et al. 1979). The satisfaction rate was 85%. The 2 patients with poor outcomes developed slight traumatic arthritis and experienced slight pain during walking.

**Table 1:** The detailed information for patients

Indexes	Detailed information
Sex	Male 9; female 4
Mechanism of injury	High falling injury 6 Road-traffic accident 7
Injury type	Closed injury
AO classification of pilon fracture	C1
Injury to operation time (average)	10 days
Fracture healing time (average)	12 weeks
Mazur classification	Excellent 9; good 2; poor 2

## Discussion

The surgical treatment of tibial pilon fractures includes open reduction and internal fixation, limited internal fixation combined with external fixation, minimally invasive treatment, and fusion of the ankle joint. According to the type of fracture and extent of soft tissue and open injury, early or delayed open reduction and internal fixation are chosen to reduce the occurrence of complications. Ankle arthroscopy may be used, along with open techniques of fracture repair. This can help to ensure normal alignment of bone and cartilage, and may also be used during fracture repair to look for cartilage injuries inside the ankle.

Sirkin et al. (2004) used open reduction and internal fixation to treat high-energy tibial fractures in a two-stage protocol according to the characteristics of the soft tissue injury. This protocol consisted of immediate open reduction and internal fixation of the fibular fracture at the early stage of injury, with simultaneous fixation of the tibial fracture using external fixators to span the ankle joint; then, anatomical reduction of the distal tibial articular surface and internal fixation were conducted after the repair of local soft tissue, to reduce the incidence of wound complications. Pollak et al. (2003) considered limited internal fixation combined with the use of external fixators to be optimal for treatment of patients with severe skeletal and soft tissue injuries; however, no advantage was shown over other methods in the treatment of patients without soft tissue injury. Japjec et al. (2013) treated 15 patients with pilon fractures (AO types C2 and C3) with fixation using external fixators and limited open reduction and internal fixation, and concluded that compared with formal open reduction and plate internal fixation, this method resulted in fewer wound complications and better ankle function.

Minimally invasive methods have been used to treat fractures according to the principles of biological osteosynthesis. Minimally invasive technology uses indirect reduction to reduce unnecessary exposure, and focuses on the management of surrounding soft tissue to protect the broken end of the fractures and surrounding blood supply, and thus improve the capacity for bony healing. Minimally invasive fixation includes percutaneous plate osteosynthesis, closed reduction and internal fixation with percutaneous cannulated screws, and arthroscopy-assisted reduction and internal fixation with percutaneous screws (Stasikelis et al. 1993; Müller and Sommer 2012; Puha et al. 2012; Tong et al. 2012; Ortmaier et al. 2015). Paluvadi et al. (2014) and Vidovic et al. (2015) used MIPO to treat distal tibial fractures, and reported good outcomes. Syed and Panchbhavi (2004) treated 7 patients with closed pilon fractures by using closed reduction and internal fixation with percutaneous cannulated screws, and reported excellent outcomes. The postoperative mean follow-up was 30.6 months, and the average score for ankle joint function was 90.8/100.

Joint fusion is rarely used in the initial treatment of fractures. It is only performed in patients with severe bone and soft tissue injury, especially those with ischemia, hypotension, multiple trauma, and serious neurological injuries (Crawford et al. 2014).

Arthroscopy and external fixators are used for the treatment of tibial pilon fractures, including AO type B and type C1-2 fractures. Cetik et al. (2007) treated a 42-year-old male patient with a tibial pilon fracture caused by high-energy trauma, with an arthroscopy-assisted unilateral external fixator and minimally invasive internal osteosynthesis. In this treatment, arthroscopy was used to reposition the fracture fragments and restore the joint surface, and the fracture fragments were fixed with screws immediately after being repositioned. The author believed that arthroscopy-assisted surgery combined with external fixator use and minimally invasive internal fixation was the optimal treatment for tibial pilon fractures, because external fixation could improve fracture alignment, arthroscopy could help restore the joint surface, and minimally invasive screws ensure fragment stability. Kralinger et al. (2003) also reported a case of closed distal tibial fracture (AO, C3) treated successfully with arthroscopy-assisted minimally invasive reduction and percutaneous screw fixation, and obtained a good outcome.

Atesok et al. (2011) published an investigation indicating that arthroscopy-assisted techniques in intra-articular fracture fixation are minimally invasive and have high accuracy, and have been successfully used for the treatment of fractures of the tibial plateau, tibial intercondylar eminence, tibial pilon, calcaneus, femoral head, glenoid, greater tuberosity, distal clavicle, radial head, coronoid, distal radius, and scaphoid. The main disadvantages of these techniques were the time-consuming and technically demanding nature of the procedures and the prolonged learning curve.

We believe that the major advantages of arthroscopy-assisted minimally invasive reduction with use of external fixators include a small wound, reduction under direct vision, and protection of the soft tissue and blood supply to the fractures. An external fixator provides indirect reduction and an effective operating space for arthroscopic implantation, especially for AO type B fractures and partial C1 fractures. This method has not been proven to be effective in the treatment of B3 and C3 fractures in clinical practice.

This study still has some limitations; for example, this method has not been compared with other traditional internal fixation methods, and few cases of tibial pilon fractures have been treated with arthroscopy-assisted minimally invasive reduction with the use of external fixators.
